# Exploring a framework for demandable services from antenatal to postnatal care: a deep-dive dialogue with mothers, health workers and psychologists

**DOI:** 10.1186/s12884-023-05722-2

**Published:** 2023-05-27

**Authors:** Chancy Mauluka, William Stones, Isabel Kazanga Chiumia, Limbika Maliwichi

**Affiliations:** 1 Kamuzu University of Health Sciences, P.O. Box 360, Blantyre, Malawi; 2 University of Malawi, P.O. Box 280, Zomba, Malawi

**Keywords:** Demand, Services, Patient Centred Care, Client Centred Care, Quality of Care, Care Practices, Antenatal Care, Postnatal Care, Labour, Delivery, Demandable Services

## Abstract

**Background:**

One of the factors affecting quality of care is that clients do not demand care practises during antenatal, intrapartum and postnatal care. This study aimed to identify care practices that can be demanded by the mother in the continuum of care from antenatal to postnatal.

**Methods:**

The study respondents included 122 mothers, 31 health workers and 4 psychologists. The researchers conducted 9 Key Informant Interviews with service providers and psychologists, 8 Focus Group Discussions with 8 mothers per group, and 26 vignettes with mothers and service providers. Data was analysed using Interpretative Phenomenological Analysis (IPA) where themes were identified and categorised.

**Results:**

During antenatal and postnatal care, mothers demanded all recommended services presented to them. Some services seen as demandable during labour and delivery included 4-hourly assessments of vital signs and blood pressure, emptying of the bladder, swabbing, delivery counselling, administration of oxytocin, post-delivery palpation, and vaginal examination. For the child mothers demanded head to toe assessment, assessment of vital signs, weighing, cord stamp and eye antiseptics, and vaccines. Women observed that they could demand birth registration even though it was not among the recommended services. Respondents proposed empowerment of mothers with cognitive, behavioural and interpersonal skills to demand services e.g., knowledge of service standards and health benefits in addition to improved self-confidence and assertiveness. In addition, efforts have to be made to address perceived or real health worker attitudes, mental health for the client and the service provider, service provider workload, and availability of supplies.

**Conclusion:**

The study found that if a mother is informed in simple language about services that she is supposed to receive, she can demand numerous services in the continuum of care from antenatal to postnatal. However, demand cannot be a standalone solution for improving quality of care. What the mother can ask for is a step in the guidelines, but she cannot probe deeper to influence quality of the procedure. In addition, empowerment of mothers needs to be coupled with services and systems strengthening in support of health workers.

**Supplementary Information:**

The online version contains supplementary material available at 10.1186/s12884-023-05722-2.

## Background

Malawi has made strides in ensuring that policies are in place to foster equity and efficiency in health care. In 2004, Malawi adopted a Health Sector Wide Approach (SWAp), with a Joint Program of Work (2004–2010) for delivery of the Essential Health Package developed in 2002. This was followed by the 2011 Malawi Health Sector Strategic Plan (HSSP I) and the revised 2017 HSSP II. The plan defines health system’s strategic objectives and priorities; the first objective of which is to increase equitable access to health and improve quality of care [1].

In 2017 the Quality Management Directorate of the Ministry of Health developed a policy, which aims to strengthen leadership, governance and social accountability to promote answerability of duty bearers (including service providers) to citizens/clients in the provision of quality services. It also hinges on improving the capacity, level of staffing, and operational efficiency in hospitals, health centres and clinics, while at the same time improving clinical practice through adherence to standards. The policy fosters client safety and people-centred care to ensure that practice responds to individual rights, preferences and values [2].

In 2014, the Reproductive Health Directorate (RHD) released service delivery guidelines to provide knowledge and direction on crucial components of reproductive health that include, among others, quality of care, counselling, maternal and neonatal health, sexually transmitted infections and harmful reproductive health practices. The document outlines recommended key care practices throughout the continuum of care from antenatal to postnatal care.

Despite these policy and programmatic efforts to improve quality, there is still fragmentation across the health sector. According to the 2016 situation analysis of the health sector in Malawi, the main challenges negatively affecting quality of healthcare revolve around weaknesses in leadership, accountability, human resource capacity, clinical practice, and insufficient client safety mechanisms and people-centred care approaches [2].

Substandard clinical practice is engendered by lack of diagnostic facilities leading to overdependence on presumptive diagnosis and treatment. The 2016 situation analysis report notes inadequate documentation and record keeping as one of the constraints to quality clinical practice explicated by inadequate use of standard operating procedures, protocols, and guidelines [2]. Further to this, there is inadequate communication between providers and clients, inconsistent use of charters with rights and responsibilities for the providers and clients, inadequate client feedback mechanisms, and limited client participation in their care.

Care practices are at the core of life-saving interventions. However, the quality of care in health centres and hospitals leaves much to be desired. For instance, the 2015–2016 Malawi Demographic and Health Survey (MDHS) reported slow progress in postnatal checks within 48 hrs after childbirth; with only 42% of mothers checked in 2016 against 41% in 2010 [3].

The 2018/9 Harmonised Health Facility Assessment (HHFA) reported that only 47% of the facilities were able to perform basic diagnostic tests and 6% of facilities had the capacity to conduct all basic diagnostic tests. Only 4% of under-five child visits reported receiving all the seven service components while on average they received 40% of the components. The components include age assessment, weighing, height measurement, plotting of weight and/or height, discussion of weight results with the mother, physical examination for the child, and counselling on feeding of a sick child.

Substantial literature has been published highlighting inadequate human resources, poor referral systems, inadequate equipment and staff attitude as major bottlenecks of quality service provision. These bottlenecks are experienced throughout the continuum of care from antenatal to postnatal care from both the demand and supply sides. There is reported global shortage of human resources for health as one of the major bottlenecks affecting quality of care [4]. In some Malawian scenarios, one midwife covers the labour, postnatal, nursery, antenatal wards and the theatre during night shift [5]. This is common in rural areas [5] where institutional births are increasingly high [3] while the number of nurse midwives remains low [6], leading to situations where “unskilled health workers are allocated to postnatal wards” [5]. Besides the shortage of human resources, inadequate supplies and low motivation contribute to poor quality of care [7–9].

However, it is imperative to note that in some cases, despite readiness of facilities to provide services, clinical practice affects quality of service. For instance, mothers are in some cases are not screened for syphilis even if the tests are in stock [10]. Such scenarios could be averted to a significant degree if communities know what to expect and demand it.

The whole study aimed to explore, develop, test and evaluate a package of interventions for improving demand for care practices. This paper addresses the specific objective of identifying care practices that can be demanded by the mother in the continuum of care from antenatal to postnatal care.

## Methodology

### Design

This was a qualitative study component of a large study that had an implementation science design. The large study aimed to explore, develop, test and evaluate and package of interventions for improving demand for care practices.

### Study setting

Data was collected at 3 levels i.e., district (District Hospital), national (Reproductive Health Department) and community (Health Centre) levels. Kaluluma Rural Hospital and Santhe Health Centre in Kasungu district were selected as intervention and comparison sites respectively. The health centres were selected on the basis that, in comparison with other health centres in the district, they had optimum availability of equipment/supplies and staff, factors that were deemed conducive for service demand.

#### Study population and sample size

The study respondents included 122 mothers, 31 health workers and 4 psychologists. While mothers and health workers were selected because they were thought to have had direct experience with care practices, psychologists were co-opted because they would help explore demandable services, which were thought to entail skills that are psychological in nature.

### Data collection

The research utilised 3 methods of data collection; namely, Vignettes Workshops, Key Informant Interviews (KIIs) and Focus Group Discussions (FGDs). The combination of methods helped to triangulate information obtained from the respondents and get further insights on thematic areas.

### Vignettes workshops to explore demandable services

The researchers facilitated simulations through which mothers and health workers created different scenarios (antenatal care, labour and postnatal care) to explore what services could be demanded and how. Eighteen mothers and 18 health workers were involved in 3 sessions of vignettes.

At the end of the vignettes, women drew and discussed prototypes demonstrating demandable services and skills for demand. In addition, the prototypes were discussed at the end of Focus Group Discussions (FGDs) with mothers and Key Informant Interviews (KIIs) with health workers and psychologists for further exploration. See Annex 2 for samples of pictorial prototypes drawn by mothers.

### Vignettes to assess compliance to standards

The researchers alongside trained data collectors facilitated simulations meant to establish the extent to which health workers complied with standards. After the vignettes, researchers asked health workers about perceptions, challenges and proposed solutions surrounding demand for care practices. Eight health workers and 8 women were involved in the simulations.

### Key informant interviews (KIIs)

The researchers alongside trained data collectors conducted open-ended in-depth interviews with different services providers and psychologists. The KIIs were conducted with 3 health workers from the district hospital, 2 officers from the Reproductive Health Directorate (RHD) at ministerial/national level, and 4 psychologists from the University of Malawi.

### Focus group discussions (FGDs)

Trained facilitators, with supervision from the researchers conducted FGDs with clients to find out about their experiences and insights on demand for care practices. A total of 48 women were involved in 8 sessions with 8 women in each session as one of the recommended numbers for Focus Group Discussions [11].

### Controlling data saturation

Data saturation was controlled by real time preliminary analysis during the data collection process. The researchers developed a grid with thematic areas to gauge the level and type of information being generated.

### Data analysis

The study utilised Interpretative Phenomenological Analysis (IPA) to identify themes from the narratives of participants [12, 13]. The first stage involved reading the transcripts repeatedly with a view to getting an overall ‘feel’. Secondly, the researchers generated themes with illustrative quotations.

### Findings

This section provides findings on perspectives of mothers, health workers and psychologists on what they thought could be the demandable services, proposed skills for demanding care, as well as perceived challenges and proposed solutions around demand. Annex 3 contains highlights of quotations from the research participants.

### Demandable services in antenatal care

During the vignettes, women demanded all the services that were introduced to them. The services included observations and clinical investigations (Blood Pressure, weight and height); physical examination (head-to-toe, Mid-Upper Arm Circumference, Fundal Height, Fetal Heart Rate and vulva inspection); laboratory investigations (haemoglobin, HIV, syphilis and urine tests); drug administration and immunisation (Iron and Folic Acid, Sulfadoxine-Pyrimethamine, Albendazole and Tetanus Toxoid Vaccine); client education/counselling (Expected Days of Delivery, diet and nutrition, diabetes, gender-based violence, and danger signs); and Long-lasting Insecticide-treated Nets.

In post-vignette discussions, both health workers and psychologists observed that all services offered during antenatal care are demandable. The same was echoed by RHD staff and psychologists during Key Informant Interviews.

“A woman can demand all assessments of physical conditions, like screening from head to toe, to assess maternal and fetal condition, size of fundus and so on. Lab services can also be demandable, like screening for syphilis…and all medications can be demanded, say SP, iron and phosphate, including the calcium, which is in the new ANC guidelines” (Midwife Nurse- Vignettes Workshop on Exploring Demandable Services).

They can demand everything…We also have issues of confidentially. During antenatal women can also demand to be seen in privacy. In addition, women can demand to be accompanied by a guardian/companion during the contacts.

(Health Worker, Reproductive Health Department-KII).

“A woman can demand those services. A certain level of literacy in needed…understanding what services are supposed to be provided.” (Organisational Psychologist-KII).

### Demandable services during labour and delivery

After being introduced to services provided during admission in labour, women demanded general examination (vital signs, blood pressure and head-to-toe), abdominal examination, auscultation, vaginal examination, urine test/examination, and monitoring the progress of labour (contractions, cervical dilation and descent).

Mothers also demanded services offered during the time between delivery and discharge (third and fourth stages). They frequently demanded administration of oxytocin, abdominal examination, checks for pulse rate, respiration and Blood Pressure, as well as vulva inspection and cleaning.

For the child, mothers demanded application of chlorhexidine digluconate on the cord stump, antiseptic/antibiotic eye drops/ointment, administration of vitamin K and vaccines against Polio and Tuberculosis, head to toe assessment, checks for vital signs, weighing, and recording of weight in the health passport. Women observed that even though birth registration was not introduced to them as one of the services offered, they could still demand it.

In addition to the services introduced to mothers, health workers said during labour and delivery women can ask for the 4-hourly assessments where the woman is checked for vital signs. Others said mothers can demand explanation of procedures and the type of labour e.g., caesarean section or forced labour.

“Women can be told that at least twice a day they are supposed to be seen to measure things like body temperature and the womb, and that they can ask for that.” (Health Worker, District Hospital- Vignettes on Exploring Demandable Services).

“The woman can also demand the type of birth positioning e.g. lie down or deliver while seated. If the pain is too much, she can ask for pain relief medicine.” (Health Worker, Reproductive Health Department-KII).

Psychologists concurred with staff from the Reproductive Health Department on the fact that a woman can demand privacy and care from a preferred service provider. It was also commonly mentioned by RHD staff and psychologists that a woman can demand pain killers.

“She could demand to have a certain person (midwife) to help her, if it’s a supportive person.” (Health Psychologist-KII).

“It depends on how the labour ward is constructed, but privacy is something women need to demand. In an ideal situation, we need private spaces for care. (RDH Staff-KII).

“I know for a fact that if the pain is too much, you can demand for pain killers. (Organisational Psychologist-KII).

However, there were occasional agreements between psychologists and health workers from the district on the idea that during the second stage of labour (descent), it is difficult for a woman to demand services and products. According to the psychologists, at this stage the woman is too traumatised to demand a service. Health workers added that apart from the trauma for the woman, it would be disturbing for them to have someone intrude into their work at this crucial moment of service provision. Psychologists proposed use of a proxy for the mother, e.g., a guardian, who could be present during delivery and demand execution of some practices omitted.

“It is difficult to demand the services especially during labour because that would be seen as unreasonable.” (Health Psychologist, KII).

“We are busy performing the procedure…for example vacuum extraction, the woman would not know what the whole process entails, and it would be disturbing on our part to have demands.” (Health Worker, District Hospital).

### Demandable services during postnatal care

As in antenatal care, women demanded all postnatal care services introduced to them. These included weighing, checks for vital signs and blood pressure, measurement of haemoglobin (Hb) level, and abdominal examination. For the child they demanded head to toe assessment, investigations on vital signs, measurement and recording of weight in the health passport, and administration of Polio vaccine.

### Skills utilised to demand services

Skills that were demonstrated and/or mentioned by the participants included: Reference to information source or past experience, call for urgent/emergency care, grievance referral/escalation, respect/politeness, assertiveness, praise, friendliness, appeal to sympathy, use of proxy/guardian and other nonverbal expressions (gestures).

#### Reference to information source or past experience

Both mothers and health workers said a woman can refer to information they heard or saw elsewhere as they demand a service. For instance, she can refer to the radio or a health talk. She can also cite an example of a service she received during her past pregnancy experience and demand a similar service.

“A woman can say… ‘last time I had my blood checked to see if it was enough, but this time you haven’t.” (Health Worker- Post Vignette Discussion).

“You can recall what the doctor said during the heath talk…you can say, ‘during the counselling you said we are supposed to be measured on the womb, but you have not done so to me.” (Mother, Kaluluma FGD).

#### Respect/Politeness

Respect was dominantly cited by women as vital for demanding a service. Some proposed gestures and conduct included kneeling, politeness in tone, maintaining social distance and avoiding public embarrassment for the health worker.

“You can show respect to the doctor by kneeling down as you ask him about something that she/he needs to do.” (Mother, Santhe FGD).

“You do not need to be too close to the doctor…that would show some disrespect.” (Mother, Santhe FGD).

“Instead of shouting out a demand, it would be important to call the doctor aside and tell him/her you have not been given something.” (Mother, Kaluluma FGD).

Occasionally, health workers observed that there is a caveat to respect and politeness. For them, women are already in a disfranchised situation whereby respect and politeness would disempowerment them further. In this regard, they suggested that respect and politeness did not have to be emphasized as a skill for demanding omitted services. Instead, they proposed that the mother should be assertive.

#### Assertiveness

In a few instances, women, health workers and psychologists noted that the mother needed to be confident that they are doing the right thing; thus, needed to be bold, especially in difficult situations.

“If the doctor is not helping you can be more emphatic to insist that he/she helps you.” (Mother, Kaluluma FGD).

“In some circumstances you need to be blunt when things are not happening. Say when you feel like you are about to deliver, and the nurse is not attending to you. You tell the nurse that you feel like something coming down, and the tone is not a normal one.” (Counselling Psychologist, KII).

However, a few health workers and one psychologist had a slightly different point of view. For them, women needed to prevent situations where insistence would lead to conflict.

“It is an exciting thing for the health worker to be reminded by a woman. We want women who are interested about their health. As long as they do not nudge you, it is fine.” (Nurse, Post Vignette Discussion).

“Whining is actually likely to backfire because it’s like the woman is challenging, okay? It’s like she’s not appreciating how busy the health worker is” (Organisational Psychologist, KII).

#### Praise and friendliness

Some mothers observed that courteous conduct did not need to be expected from health workers only. Thus, women could use praise as verbal incentives to induce a health worker towards providing omitted care practices. They also observed that their friendliness towards the health workers would influence care.

“It is not only the responsibility of health workers to be friendly. They are also humans who can be influenced to do something if someone is friendly to them.” (Mother, Kaluluma FGD).

“You can acknowledge some good things that the doctor has already done to you, and then remind him/her of the others that you think have been missed.” (Mother, Santhe FGD).

“A woman can acknowledge the workload of the nurse before requesting for something. For instance, you can say: ‘I know you are very busy, but I am interested to know how my labour was…would you help me?” (Mother, Post Vignette Discussion on Exploring Demandable Services).

#### Appeal to sympathy and call for urgency

Mothers said to drive a health worker towards agile provision of a service, they could use bodily or emotional expressions that would demonstrate pain or concern about a condition.

“In my walk and facial expression, I showed that I was feeling pain in my body. In that way the doctor would help me quickly.” (Woman, Post-Vignette Discussion on Exploring Demandable Services).

“The tone has to show that you are really worried about something.” (Woman, Kaluluma FGD).

“It might be that you have talked for quite some time and do not want the doctor to think you are difficult. You can just do a gesture for it. For example, try to breastfeed your child and make a mistake…show you are struggling. She can start advising you about breastfeeding” (Woman, Kaluluma FGD).

However, this was contrary to what psychologists thought. For them mothers should avoid use of any emotionally manipulative or deceptive tactics to get attention. Instead, they need to express their needs directly.

“Touching the tummy in pain - if you are pretending that you are feeling the pain, sometimes it could be noticed so it’s sort of breaking the trust between you and your health provider.” (Health Psychologist, KII).

“Crying may work maybe once in a while but it’s not an effective strategy… they might just feel like you are just being dramatic and attention seeking.” (Developmental Psychologist).

“They should be able to explain to the health worker that this is what I am going through so I was wondering if it could be possible if… not even in a pleading way.” (Health Psychologist-KII).

#### Grievance referral/escalation

Health workers from the Reproductive Health Department (RHD) pointed out that women could also demand care using a system of complain mechanism through which they could channel their unmet needs. The office of the hospital ombudsman could play the role of grievance reception and address. Psychologists concurred with the RHD officers on this observation.

“Apart from the ombudsman, women can channel their demands through the departmental heads in charge. This means they need to be oriented about the focal person or supervisors they can talk to should they feel certain services are not being offered.” (RDH Officer, KII).

“Make them aware of what they expect, and if they see or experience anything to the contrary, there has to be mechanisms on how these women can report.” (Organisational Psychologist, KII).

### Factors influencing demand

Respondents noted various factors that can motivate or demotivate a woman to demand a service. These comprised knowledge of service standards, perception of health benefits, perceived or real health worker attitude, mother’s self-confidence, fear of side effects and diagnostic results, cultural beliefs and norms, mental health for the client and service provider, service provider workload, availability of supplies, and child-centred attitude in postnatal care.

#### Knowledge of service standards

All respondents indicated that women could demand services if they knew the care practices that are expected at the health centre. They need basic knowledge in simple language to understand the service standards.

“For most mothers growth monitoring and promotion is about weighing. They don’t mind that there are other services like head-to-toe assessment, BP etc. They need to know about the other services and their importance.” (Kaluluma Health Worker, Post Vignette Discussion on Observation of Care).

“The problem is that sometimes we do not know what we are supposed to get at the health centre…if we know we can ask.” (Mother, Kaluluma FGD).

“A certain level of literacy is needed…understanding the services that are supposed to be provided to you…and you can demand those services.” (Organisational Psychologist, KII).

#### Perception of health benefits

Mothers said they would be motivated to demand on the basis that they need to prevent infections and morbidities for themselves and their child. Thus, they would be compelled to ensure the health worker delivers quality service.

“You can ask for an injection to stop losing too much blood because when you are losing too much blood, you can die.” (Woman, Kaluluma FGD).

“When we have not been given iron tablets, the child might be born with infections…The health worker is busy …but it is your life, therefore you have to help him help you.” (Woman, Santhe FGD).

#### Mothers’ self-confidence

All the respondents identified fear as an element that would impact the woman’s ability to demand certain services. According to the psychologists, the fear might be rooted in lack of self-confidence. A mother’s perception is at times that the health workers are authorities and experts in their field, as such they cannot be told what to do. Such was correspondingly observed by mothers and health workers.

“Most of the women especially in the rural settings would largely be illiterate or semi-illiterate and therefore when they are in the presence of someone who has been to school and has everything else and is in that uniform, they would obviously freeze and would not ask. That’s quite a challenge.” (Organisational Psychologist, KII).

“They went to school. They know what they are doing, so you cannot ask them for something.” (Mother, Kaluluma FGD).

#### Fear of side effects and diagnosis results

Both women and health workers observed that it would be difficult for some women to demand Tetanus Toxoid Vaccine (TTV) and Sulfadoxine-Pyrimethamine (SP) for malaria prevention. This was because pregnant women resent the two products owing to fear of what they think are side effects of the products during pregnancy. Psychologists on the other hand noted that demand for HIV testing maybe hindered by fear of positive results.

“They say Fansidar (SP) causes dizziness and it affects the fetus. They will insist that they have not taken any food and do not want to take medicine on direct observation, when they actually want to throw it (the SP) away.” (Health Worker, Santhe, Post Vignette Discussion on Observation of Care).

#### Cultural beliefs and norms

Psychologists noted that personal beliefs and culture also affect women’s ability to demand certain services. For instance, in some cases, due to cultural beliefs about what the role of a woman is with regards to childbirth, some women might have the mentality that asking for pain medication is not recommendable. For others, the limitation would come about because of the influence of culture on their ability to feel free with a medical practitioner of the opposite sex.

#### Mental health for the client and service provider

Psychologists observed that Malawi does not offer preconception counselling which is important for preparing mothers who are planning for pregnancy. For them, limited psychological and emotional support is the crux of challenges that both mothers and health workers face. That can be a significant hindrance to the optimal provision of quality and demand for it.

“Mental health is lacking… it’s a very important aspect, if it could be incorporated… mental health is not only about depression but even the issue of self-image and stuff like that.” (Health Psychologist, KII).

#### Child-centred attitude in postnatal care

Some health workers said they usually do not provide services for the mother after she gives birth as the attention is now drawn towards the child. Mothers equally said their concentration is on the child and not themselves. That perception hinders willingness to demand omitted services for the mother.

“Mostly we ask the mother if she is breast feeding, but you can see that the concern is much on the nutrition and the health of the child.” (Health Worker, Post Vignette Discussion).

“We do not expect much as a mother, unless we feel sick…what we want to see is that the child is vaccinated, and they have taken the weight.” (Mother, Health Worker Post Vignette Discussion).

### Suggestions for improving demand for services

Respondents suggested elements that would facilitate demand for services from clients. These were thought to be important prerequisites from both the demand and the supply side. The suggestions hinged on orientation/training of mothers and health workers coupled with provision of supplies for health.

#### Orientation/Training for mothers and health workers

All the respondents noted that women need to be sensitised about demandable services. While psychologists and health workers added that women need training on their health rights and obligations to demand, health workers observed that in addition, women have to know the importance of those care practices. Psychologists on the other hand added that such training has to include mental health to enable the woman handle stress in pregnancy and to help the health worker address stress in the workplace; thus, creating a conducive environment for the two to communicate effectively.

Psychologists equally observed that both the health worker and the mother need training in interpersonal communication. While the mother needs skills to communicate assertively, politely and without fear, the health worker needs skills in respectful maternity and hospitality. This resonated with responses from mothers who indicated that they need assurance from health workers that they can demand care practices. Such assurance would be expressed in words, actions and gestures from the health workers. For instance, health workers need to clearly state that they need support from women to help them do their job well.

“They have to know the service charter... mothers should know if they come to the hospital what has to be demanded…the services that we can offer to them e.g., dishing leaflets… have posters to be pasted in the community and health centres so they know.” (RDH Staff, KII).

“If everybody is demanding for the same doctor, that person will be overwhelmed, and they might end up being stressed. Health workers need stress management skills.” (Health Psychologist, KII).

“The way they conduct themselves…the way they walk…it should show that they have umunthu (‘humanity’). When someone is coming from that side, you should actually feel you can talk to them. That is nsangala zodutsa (‘friendliness by gesture/countenance’).” (Mother, Santhe FGD).

#### Provision of supplies

Both health workers and psychologists noted that it is the responsibility of management to ensure availability of supplies across the continuum of care as chronic unavailability of supplies is likely to reduce the mother’s motivation to demand for a service.

“If a mother is told that she cannot be helped because a service is not available, she will unlikely demand it the next time she comes.” (Health Psychologist, KII).

## Discussion

Since the 1960’s feminist movements have advocated for reforms in reproductive rights [14] and gender equality /equity activists have often utilized feminist approaches to garner women’s empowerment in all aspects of life including health [15, 16]. In these approaches, the notion of power sharing and consciousness raising is deemed vital to empowerment [17–19]. In his book, Pedagogy of the Oppressed, Paulo Freire discusses the process of empowerment through critical consciousness where the ‘custodian of knowledge’ and the learner engage in a dialogue of reflective thought and correlative action in a mutual relationship that demystifies knowledge [20].

Taking a stance of empowerment, several studies have been conducted to analyse how power relations are perceived and should be managed between patients/clients and health workers. There is a general observation that sharing power entails aspects of sharing clinical information with clients, making mutual decisions about care [21] and establishing rapport [22]. Research has established that clients appreciate care when it is brokered by active listening from providers, use of local language, counselling, provision of information on treatment options, as well as clarity of prescription and next appointment/s [23]. While acknowledging that clients need information and participation in clinical decision making, this research went further, investigating what kind of information would be practically needed, what services would practically be demanded and how such services would be demanded by the client in the continuum of care. The research contributed to clinical practice by establishing a minimum package of services that can be practically demanded by the client. Refer to Annex 1 for a Proposed Minimum Package of Demandable Services.

Observations from respondents pointed to the fact that there are socioecological factors that influence demand for services at the point of care. As categorised by the Socioecological Model of Behaviour, the factors ranged from personal, interpersonal, community, institutional and societal levels [24]. At personal level the findings resonated with the skills taxonomy by Danish, Forneris, Hodge, and Heke, who categorised life skills as behavioural (communicating effectively with others), cognitive (making effective decisions), interpersonal (being assertive), and intrapersonal (setting goals)” [25].

What can be seen as cognitive and intrapersonal skills are scenarios where mothers made reference to information sources or past experiences and called for urgent/emergency care. Interpersonal skills were seen in propositions/demonstrations of assertiveness. Finally, mothers demonstrated and/or proposed behavioural skills that included respect/politeness, praise, friendliness, appeal to sympathy, and use of grievance referral/escalation. Such skills need to be explored with mothers to enhance demand for care practices.

While some skills may be taught cognitively e.g., verbal communication skills in phrasing a demand, others can be learnt socially through sharing of experiences and simulations e.g., emotional expressions and gestures. This is in agreement with the social cognitive theory of behaviour which stipulates that individuals learn through observation and emulation [26]. Studies have shown that peer education through trusted mothers yields impact in improving client practices for improving maternal and neonatal health [27]. For Demandable Services such approaches can be adopted to ensure that mothers have knowledge of care practices in simplified messages.

Counselling for skills needs to build on cultural resources available to the client [28]. It was clear from responses of this study that skills are rooted in culture e.g., respondents referred to cultural values, like respect, which is demonstrated in various ways perceived appropriate by the client and the provider. Therefore, skills’ building for Demandable Services has to be contextualised within specific cultural values in which they function. This entails that there can hardly be a standardisation of skills and values around skills for demand.

However, knowledge and skills are not enough as a driver of women’s behavioural choices in maternal health. Women require agency to speak up and act [29, 30]. Unfortunately, in the context of Demandable Services, we see the mother deterred by fear that stems from a general lack of self-confidence and unknown/perceived repercussions of demand. Besides the life skills competence, individual agency comes into play as the mother has to believe she has the right and responsibility to speak up and act for the benefit of her and her child’s health. This implies that developing a package of empowerment for mothers needs to seriously consider aspects of individual agency.

At the community level, we see demand being shaped by cultural norms. For instance, when a woman is assertive, she may be viewed as being rude. This would create an environment where the mother mildly demands a service or skips it if she does not have elevated individual agency. For others, the limitation comes about because of the influence of culture on their ability to feel free with a medical practitioner of the opposite sex [31]. This entails that for demand to be sustainable there has to be communal social accountability initiatives that establish demand as a norm. Such initiatives have to capsulate broad pro-gender dimensions aimed at unleashing the power in women.

Social accountability significantly contributes to visible outcomes in services provision [32–35]. In Ethiopia a social accountability project empowered community members with knowledge on service standards and created dialogue between communities and service providers on addressing bottlenecks to quality. The results showed significant improvements in the percentage of women who had their blood pressure measured and blood urine tested during the last pregnancy [36]. In Malawi a social accountability project used citizen forums called Bwalos, interactive theatre for development and community radio to create dialogue between right holders (communities) and duty bearers (health workers, administrators, and politicians) in ensuring availability of supplies, equipment and commodities for improving Maternal, Newborn, Child and Adolescent Health (MNCAH). The project created intermediate outcomes that included improvement in referral systems, water and sanitation facilities, staffing, power supply (electricity), drugs and commodities, and space in health facilities [37]. Similar interventions, coupled with empowerment of individual mothers in demandable services, can catalyse changes in service provision.

However, even in the presence of individual willingness and an enabling cultural environment, institutional bottlenecks affect provision of services [38–41]. Researchers have found that in many contexts health worker attitudes are characterised by undignified behaviour and lack of emotional support for the client; scenarios that are thought to be influenced by stress, fatigue, workplace frustration [39, 41–44] and asymmetrical power relations that make health workers dominate the interaction [41, 45] and the woman disfranchised [46]. In the context of Demandable Services, while on the one hand there is there is the actual lack of hospitality among some health workers, on the other hand, attitudes of mothers towards health workers also contribute to their own apathy towards demand. This demonstrates that for demandable services to thrive, there has to be dialogue between the supply side and the demand side to create rapport and address any perceived or real barriers.

**Fig. 1:**
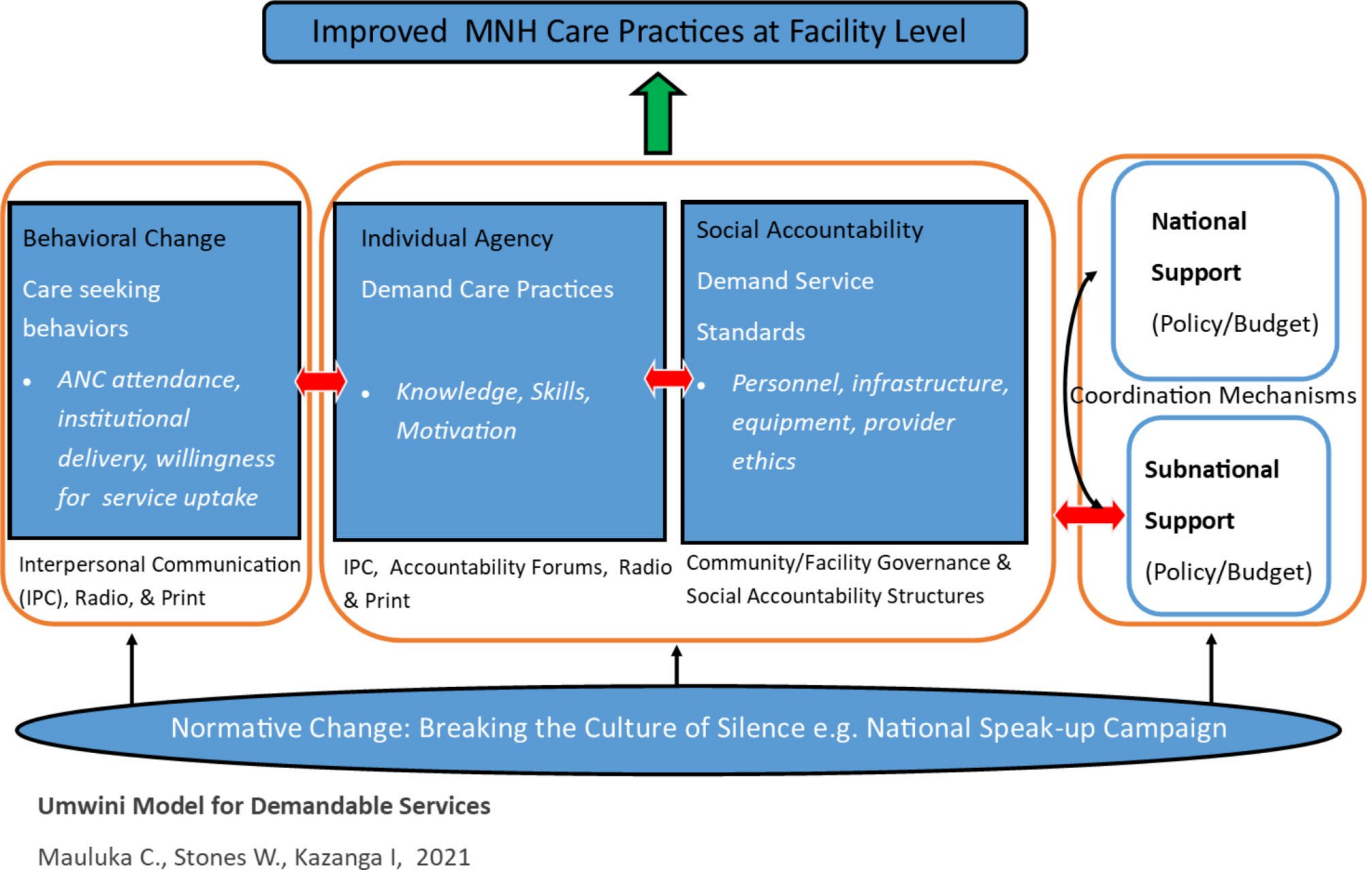
A Proposed Framework for Demandable Services from Antenatal to Postanal Care

The model in Fig. 1 below illustrates a proposed framework for Demandable Services in maternal and neonatal health; termed as Umwini Model owing to the unique emphasis on individual power and sense of ownership (umwini) for one’s health as a drive of demand.

Related to acceptability and willingness, studies have found that for effective rollout, neonatal and maternal clinical practices need to be accepted by both the provider and client [47, 48]. Demand as a new or enhanced practice relates to the same phenomenon where it needs to be accepted by the provider and the client. The positive element is that mothers and health workers commonly accepted the new phenomenon/practice to have women actively demand quality. However, fears from a few health workers, that women would become unmanageable, need to be taken into consideration. This implies that rolling out an intervention on Demandable Services requires establishing a situation of rapport among clients and service providers to ensure they do not see one another as warring factions, but rather as collaborators.

However, there is a limitation in client demand as a contribution to quality of care. While women can only demand tipping points for a care practice i.e., merely asking the service provider to observe a crucial step in service provision, the quality of procedure will still depend on the service provider.

## Study limitations

Vignettes as a research method is dramatization of life. Research participants might not have presented themselves in a way they would in real life. Though widely used to resolve the challenge of research time and costs, a closer to life or real-life methodology of observation would have been more informative. One notable challenge encountered with vignettes was that in some cases, during observation of care, the trained women became excessively demanding. While on the positive side it enabled the researchers to further understand what services women could demand and how they could demand the services, on the downside such occasions did not create enough opportunity for the research to learn about the services that would have been missed by the service providers. Future research endeavours can explore utilising untrained women during observation of care.

In addition, the sample was not representative of the population of women in Kasungu district or Malawi. Therefore, the proposed package of demandable services that stemmed from this study can only be applied elsewhere with a caution to further observe and make possible alterations.

The research did not have a deep dive into demandable services during the second stage of labour (from descent to removal of placenta). This was because originally it was envisaged that this is a difficult stage to engage mothers into demand for services. Therefore, in attempt to narrow down the scope, this stage was not included as part of vignettes. However, it was covered during KIIs with health workers and psychologists and generally there were pointers to the fact that services at this stage can also be demanded.

There is overwhelming evidence that maternal conditions and behaviour will affect birth outcomes [49]. On the other side there are compelling observations that paternal conditions and behaviours also contribute to health pregnancy e.g., blood group [50] and psychosocial and economic support [51]. This research, however, did not involve men in exploring demandable services. This was mainly because participation of men in antenatal, delivery and postanal care is almost non-existent in Malawi. The first step would be to motivate men to get involved before exploring demandable services with them. Future research will need to explore perspectives of men in demandable services. However, the first step requires soliciting their participation in the continuum of care.

## Conclusion

The study showed that each of the services offered during antenatal and postnatal care can be demanded by the mother, while most of the services offered during labour and delivery are demandable. There are a number of cognitive, behavioural and interpersonal skills that can be utilised by mothers to demand e.g., reference to information source or past experience, call for urgent/emergency care, grievance referral/escalation, respect/politeness, assertiveness, praise, friendliness, use of proxy/guardian and other sundry nonverbal expressions (gestures) internal to the client’s culture.

Socioecological factors need to be considered when creating demand for care practices. These include knowledge of service standards, perception of health benefits, perceived or real health worker attitude, mother’s self-confidence, cultural beliefs and norms, mental health of the client and the service provider, service provider workload, and availability of supplies.

However, demand cannot be a standalone intervention for improving quality of care. What the mother can ask for is a step in the guidelines, but she cannot delve deep to influence quality of the procedure. Further research is needed to explore the extent to which empowered mothers can demand care practices and if such demand can generate results in terms of actual service provision during labour, delivery and postnatal care.

## Electronic supplementary material

Below is the link to the electronic supplementary material.

**Additional file 1:** Annex 1 and 2

**Additional file 2:** Annex 3

## Data Availability

Most data generated or analysed during this study is included in this published article. Additional data can be accessed upon request by writing to chancymauluka77@gmail.com/m201980015215@stud.medcol.mw.

## Declarations

### Compensation

Participants did not receive special compensation for participating in the research. However, they received transport reimbursement and were served with refreshments after the discussions.

### Disclaimer

The information in the document expresses views and opinions of the authors and do not represent UNICEF’s position.

## Abbreviations

APGAR: Appearance, Pulse, Grimace, Activity and Respiration
ANC: Antenatal Care
BCG: Bacillus Calmette-Guerin
BP: Blood Pressure
EDDs: Expected Days of Delivery
EENT: Ears, Eyes, Nose, Throat
EPPM: Extended Parallel Process Model
EWEC: Every Woman Every Child
FGD: Focus Group Discussion
FH: Fundal Height
FHR: Fetal Heart Rate
Hb: Haemoglobin
HHFA: Harmonised Health Facility Assessment
HSA: Health Surveillance Assistant
HSSP: Health Sector Strategic Plan
IDI: In-depth Interview
IFA: Iron-Folic Acid
IPA: Interpretative Phenomenological Analysis
KII: Key Informant Interview
LLI: Long Lasting Insecticide-Treated Net
MDHS: Malawi Demographic and Health Survey
MNCAH: Maternal, New-born, Child and Adolescent Health
MNH: Maternal and Neonatal Health
MUARC: Mid-Upper Arm Circumference
PNC: Postnatal Care
RHD: Reproductive Health Directorate
SP: Sulfadoxine-Pyrimethamine
Swap: Health Sector Wide Approach
TTV: Tetanus Toxoid Vaccine
