# Supplementary material for: Exploring a framework for demandable services from antenatal to postnatal care: a deep-dive dialogue with mothers, health workers and psychologists
Source: BMC Pregnancy Childbirth. 2023 May 27;23:390. doi: 10.1186/s12884-023-05722-2 (PMC10224227; doi:10.1186/s12884-023-05722-2)
Supplement: Supplementary file 1 — Additional file 1: Annex 1 and 2 [file 12884_2023_5722_MOESM1_ESM.docx]

**Annex 1: Minimum Package of Demandable Services**

**ANC Demandable Services**

During ANC a mother can demand clinical investigations/observation (BP, weight and height), physical examination (head-to-toe, MUARC, FH, FHR and vulva inspection), laboratory investigations (Hb, HIV, syphilis and urine tests), drug administration and immunisation (IFA, SP, Albendazole and TTV), client education/counselling (EDDs, nutrition, diabetes, gender-based violence, and danger signs) and an LLIN for malaria prevention.

**Demandable Services during Labour and Delivery**

During the first stage of labour (onset to descent) the mother can demand 4-hourly assessments for vital signs (temperature, heart/pulse rate and respiration) and blood pressure. Other services she can demand include head-to-toe assessment, abdominal and vaginal examination, urine test and assessment of the progress of labour (cervical dilation etc).

During the second stage (descent), the woman maybe in a traumatic state to demand care practices. However, with the aid of a guardian it is possible to have some services demanded. This could be a trend towards ‘participatory midwifery’ where the client and the guardian could be well informed about the importance of each practice and the procedures involved. Pre-delivery rapport among the trio (nurse, guardian and the client) could create a conducive environment for the guardian to hint on a skipped practice during delivery.

Some practices that can be demanded in preparation for delivery include emptying of the bladder, swabbing (thighs and vulva) with antiseptic solution, vaginal examination, delivery counselling, continuous attendance by midwife, and position of delivery.

During actual delivery, the guardian can remind the midwife about helping the woman to rest between contractions, offering encouraging feedback after each push, reminding the mother to pant after crowning, quickly checking the cord around the neck, cleaning secretions from eyes, mouth and nostrils with sterile swab, discarding perineal pad in time, attachment (placing baby on mother’s abdomen/chest skin to skin) and warming (covering baby and mother with blanket). The guardian can equally ask if the baby’s appearance, pulse, grimace, activity and respiration (APGAR) are normal and demand palpation to rule out presence of additional baby(ies).

From the removal of placenta to the time of discharge (third to fourth stage), the mother can demand checks for temperature, respiration, heart rate, abdominal and vaginal examination, and administration of oxytocin. For the child she can demand head to assessment, checking heart rate and temperature, weighing and recording of weight in health passport, and administration of antiseptic ointment/antibiotic for eyes, vitamin K, vaccines (BCG & Polio) and antiseptic for the cord stump.

**PNC Demandable Services**

During Postnatal Care (PNC) the mother can demand weighing, checks for BP and Hb as well as vaginal and abdominal examination. For the child she can demand checks for vital assigns, head to toe assessments, weighing followed by recording of weight in the heath passport, and administration of Polio vaccine if not given at birth.

**Annex 2: Samples of pictorial prototypes by mothers**


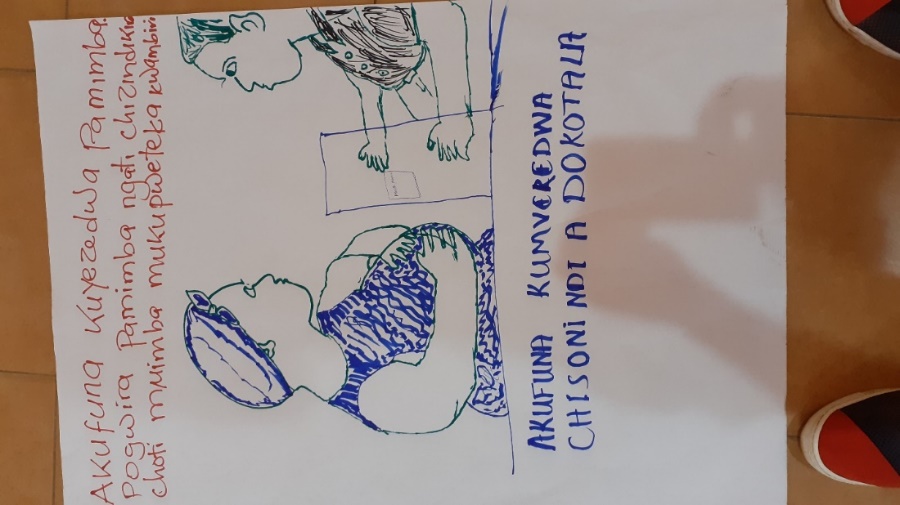


**Top Text:** She wants her womb to be checked. She touches it as a sign that she is feeling severe pain

Bottom Text: SHE IS SEEKING SYMPATHY FROM THE DOCTOR


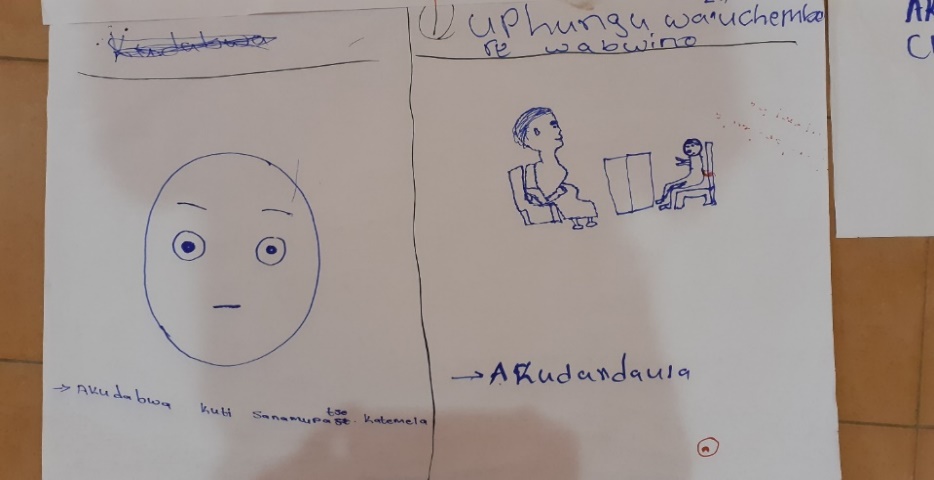


**Left Picture and Text:** She is surprised that she has not been given vaccination (meaning vaccination for the child)

**Right Picture and Text:**

*Top Text:* Counselling on healthy pregnancy

*Bottom text:* She is complaining


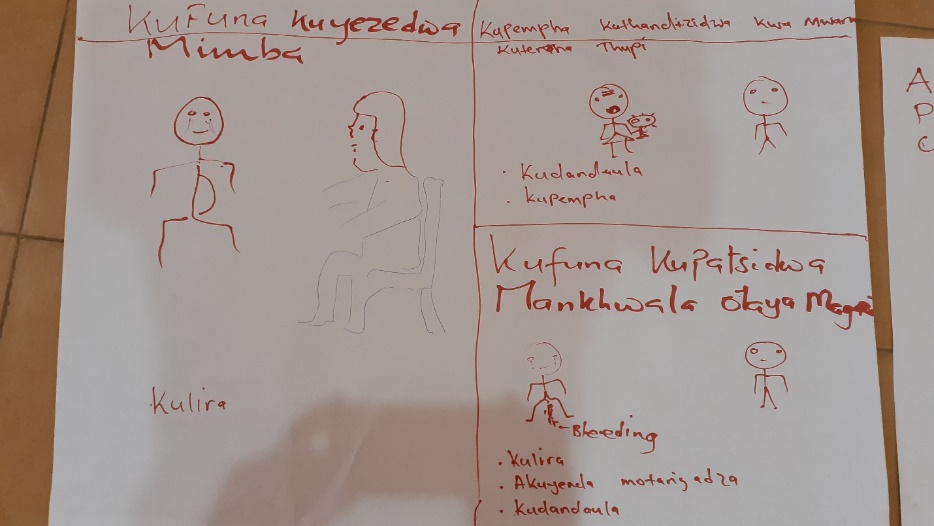


**Left Picture and Text:**

*Top Text:* She wants her pregnancy to be checked/tested

*Bottom text:* She is crying

**Right Picture and Text:**

*Picture and Text on Top:* She’s seeking help for her child with fever……She’s complaining…She’s pleading

*Picture and Text on bottom:* She want medicine that reduces bleeding…She’s crying…she’s walking with difficulties…She’s complaining
