# Supplementary material for: Exploring a framework for demandable services from antenatal to postnatal care: a deep-dive dialogue with mothers, health workers and psychologists
Source: BMC Pregnancy Childbirth. 2023 May 27;23:390. doi: 10.1186/s12884-023-05722-2 (PMC10224227; doi:10.1186/s12884-023-05722-2)
Supplement: Supplementary file 2 — Additional file 2: Annex 3 [file 12884_2023_5722_MOESM2_ESM.docx]

**Annex 3: Highlights of Quotations from Mothers, Providers and Psychologists**

**Annex 3.1: Demandable Services in Antenatal Care**

| **Mothers** | **Providers** | **Psychologists** |
| --- | --- | --- |
| “A woman can demand check-up for blood pressure…she also can demand to be weighed” (Mother, FGD, Santhe)  “You can ask to be weighed every time you for to the clinic during pregnancy.” (Mother, FGD, Santhe)  “The mother can demand a net, iron tablets and Fansidar”  “We know we are supposed to receive a bed net. We can ask for it if we are not given.”  “The tablets that increase blood in the body. We can ask for that when we go for the antenatal clinic…but also blood pressure. They need to measure blood pressure...not forgetting the medicine that prevents malaria.” (FGD, Mother, Kaluluma)  “We can demand when we have not been given vaccination or when we have not been tested for HIV...and iron tablets.” (FGD, Mother Kaluluma)  “You can ask for tetanus vaccine also.” (FGD, Mother, Santhe)  “All the services can be demanded …if you know what the doctor needs to give you.” | “A woman can demand all assessments of physical conditions, like screening from head to toe, to assess maternal and fetal condition, size of fundus and so on. Lab services can also be demandable liked screening for syphilis…and all medications can be demanded, say SP, iron and phosphate, including the calcium, which is in the new ANC guidelines” (Midwife Nurse- Vignettes Workshop on Exploring Demandable Services.)  “They can demand everything…We also have issues of confidentially. During antenatal women can also demand to be seen in privacy. In addition, women can demand to be accompanied by a guardian/companion during the contacts.” (HD Staff-KII). | “For every antenatal visit they can demand weight, blood pressure, blood test, urine tests, the foetal heartbeat, the fundal height, height of the child… depending on what stage, estimated foetal weight, EDD” – Health Psychologist  “In terms of the actual services, obviously they get demand checks, BP, blood levels… I think iron levels as well” – Organisational Psychologist  “At an antenatal appointment, a woman can demand that her blood pressure be checked…get sometimes a sample of urine to check different things whether they are have high blood pressure problems or diabetes problems.” – Counselling Psychologist |

**Annex 3.2: Demandable Services during Labour and Delivery**

| **Mothers** | **Providers** | **Psychologists** |
| --- | --- | --- |
| “You can ask for an injection to stop losing too much blood” (Mother FGD, Kaluluma)  “You can also ask to be operated on when you are not giving birth.” (Mother FGD, Kaluluma)  “You can ask that the nurse checks your vaginal path, if it is getting ready for delivery.” (Mother FGD Santhe)  “They also need to give you water to drink. You can ask for water. You also can ask if it is appropriate to be put on a drip (IV)” ( Mother, FDG, Santhe)  “ The woman can ask for birth registration of the child We are told these days that the child has the right to be named and be registered immediately after birth.” Kauluma | “The woman can also demand the type of birth positioning e.g., lie down or deliver while seated. If the pain is too much, she can ask for pain relief medicine.” Health Worker, Reproductive Health Department-KII.  “Women can be told that at least twice a day they are supposed to be seen to measure things like body temperature and the womb, and that they can ask for that.” Health Worker, District Hospital- Vignettes on Exploring Demandable Services.  “It depends on how the labour ward is constructed, but privacy is something women need to demand. In an ideal situation, we need private spaces for care” RDH Staff-KII.  “We are busy performing the procedure…for example vacuum extraction, the woman would not know what the whole process entails, and it would be disturbing on our part to have demands.” Health Worker, District Hospital. | “She could demand to have a certain person there, if it’s a supportive person.” Health Psychologist-KII.  “I know for a fact that if the pain is too much, you can demand for pain killers.” Organisational Psychologist-KII.  “Depending on the resources available in that hospital, even asking for privacy is something that the woman can demand, can the curtain be closed” – Counselling Psychologist  “It is difficult to demand for the services especially during labour because they are seen as unreasonable” Health Psychologist.  “When a woman has delivered a baby, she goes through… shock and exhaustion… that would then make it difficult for her to open up and come forward” Counselling Psychologist  “The woman needs to know whether they are ready to go home and what sorts of care they should expect before going home and even when they go home, how do they take care of themselves… hygiene, diet, breastfeeding” – Organisational Psychologist |

**Annex 3.3: Postnatal Demandable services**

| **Mothers** | **Providers** | **Psychologists** |
| --- | --- | --- |
| “She can ask for vaccination of the child”  “Reminding the doctor for to weigh your baby.”  “A woman can request that the child be weighed and can ask for breastfeeding tips.” | “All services that are in the reproductive health guidelines can be demanded by the woman if they are not offered.”  “A woman can demand head to toe assessment for the child and she can ask the nurse to check her, especially if she has problems. For example, if she has breastfeeding problems.” | “All the services can be demanded. What is important also is that the woman should ask for interpretation of results and advise. For instance, when the growth monitoring card is filled by the health worker, she should demand interpretation of that and what she neds to do thereafter.” |

**Annex 3.4: Skills Utilised to Demand Services**

| **Mothers** | **Providers** | **Psychologists** |
| --- | --- | --- |
| “You can recall what the doctor said during the heath talk…you can say, ‘during the counselling you said we are supposed to be measured on the womb, but you have not done so to me.” Mother, Kaluluma FGD.  “Instead of shouting out a demand, it would be important to call the doctor aside and tell him/her you have not been given something.” Mother, Kaluluma FGD.  “You can show respect to the doctor by kneeling down as you ask him about something that she/he needs to do.” Mother, Santhe FGD.  “You do not need to be too close to the doctor…that would show some disrespect.” Mother, Santhe FGD.  “People know you are respectful through the way you speak. You have to show that you are not being rude, but you are requesting.” Mother, Kaluluma FGD.  “If the doctor is not helping you can be more emphatic to insist that he/she helps you.” Mother, Kaluluma FGD.  “It is not only the responsibility of health workers to be friendly. They are also humans who can be influenced to do something if someone is friendly to them” Mother, Kaluluma FGD.  “You can acknowledge some good things that the doctor has already done to you, and then remind him/her of the others that you think have been missed.” Mother, Santhe FGD.  “A woman can acknowledge the workload of the nurse before requesting for something. For instance, you can say: ‘I know you are very busy, but I am interested to know how my labour was…would you help me?” Mother, Post Vignette Discussion on Exploring Demandable Services.  “It might be that you have talked for quite some time and do not want the doctor to think you are difficult. You can just do a gesture for it. For example, try to breastfeed your child and make a mistake…show you are struggling. She can start advising you about breastfeeding” Woman, Kaluluma FGD.  “In my walk and facial expression, I showed that I was feeling pain in my body. In that way the doctor would help me quickly.” Woman, Post-Vignette Discussion on Exploring Demandable Services.  “The tone has to show that you are really worried about something.” Woman, Kaluluma FGD.  “You may not just say ‘check my womb’…A woman can say I am feeling like pain in my womb. That would make the health workers check the womb.” Mother, Post-Vignettes Discussion. | “A woman can say… ‘last time I had my blood checked to see if it was enough, but this time you haven’t.” Health Worker- Post Vignette Discussion.  “Women are usually not confident and are fearful…We need to look carefully at respect. I think it may further reduce their power to demand. They need to show some confidence” Health Worker, Kaluluma, Post Vignette Discussion.  “It is an exciting thing for the heat worker to be reminded by a woman. We want women who are interested about their health. As long as they do not nudge you, it is fine.” Nurse, Post Vignette Discussion.  “Apart from the ombudsman, women can channel their demands through the departmental heads in charge. This means they need to be oriented about the focal person or supervisors they can talk to should they feel certain services are not being offered.” RDH Officer, KII. | “In some circumstances you need to be blunt when things are not happening. Say when you feel like you are about to deliver, and the nurse is not attending to you. You tell the nurse that you feel like something coming down, and the tone is not a normal one.” Counselling Psychologist, KII.  “Politeness is a gesture that can motivate someone to offer a service, but women should know that they are not bound by it.” Developmental Psychologist, KII.  “Whining is actually likely to backfire because it’s like the woman is challenging, okay? It’s like she’s not appreciating how busy the health worker is” – Organisational Psychologist.  “They should be able to explain to the health worker that this is what I am going through so I was wondering if it could be possible if… not even in a pleading way.” Health Psychologist-KII.  “Crying may work maybe once in a while but it’s not an effective strategy… they might just feel like you are just being dramatic and attention seeking.” Developmental Psychologist.  “Make them aware of what they expect, and if they see or experience anything to the contrary, there has to be mechanisms on how these women can report” Organisational Psychologist, KII.  “Touching the tummy in pain - if you are pretending that you are feeling the pain, sometimes it could be noticed so it’s sort of breaking the trust between you and your health provider.” Health Psychologist, KII. |

**Annex 3.5: Factors Influencing Demand for Care Practices**

| **Mothers** | **Providers** | **Psychologists** |
| --- | --- | --- |
| “The problem is that sometimes we do not know what we are supposed to get at the health centre…if we know we can ask.” Mother, Kaluluma FGD.  “When we have not been given iron tablets, the child might be born with infections…The health worker is busy …but it is your life, therefore you have to help him help you.” Woman, Santhe FGD.  “We are sometimes shy and scared to ask for something.” Mother, Santhe FDG.  “They went to school. They know what they are doing, so you cannot ask them for something.” Mother, Kaluluma FGD.  “We do not expect much as a mother, unless we feel sick…what we want to see is that the child is vaccinated, and they have taken the weight.” Mother, Kauluma Post Vignette Discussion.  “How the doctor looks can make you ask for something or not.” Mother Santhe FGD  “The way the doctor is caring about you….If the doctor is on the phone you cannot ask for something.” Kaluluma FGD | “For most mothers growth monitoring and promotion is about weighing. They don’t mind that there are other services like head-to-toe assessment, BP etc. They need to know about the other services and their importance” Kaluluma Health Worker, Post Vignette Discussion on Observation of Care.  “They say Fansidar (SP) causes dizziness and it affects the fetus. They will insist that they have not taken any food and do not want to take medicine on direct observation, when they actually want to throw it away.” Health Worker, Santhe, Post Vignette Discussion on Observation of Care.  “There are many women and a few health workers. It is difficult to have time dedicated for each woman.” Health Worker, Santhe Post Vignette Discussion.  “If the woman demands that she be tested for blood levels but…for example we do not have hemocue machine then we cannot. Availability of equipment is crucial.” Kaluluma, Post Vignette Discussion | “The problem is…not knowing what is supposed to happen so that they should even say that ‘you know that this hasn’t happened (yet)’.” – Developmental Psychologist  “A lot of women know about blood test but they may not also ask about it because they are afraid of finding out whether they are HIV positive or not” – Developmental Psychologist  “Personal beliefs that this is something motherhood is, I have to suffer, I have to go through this pain” – Counselling Psychologist  “In certain cultures, you might not discuss with the opposite sex with certain issues because your culture don’t (sic) allow you to do that.” – Organisational Psychologist  “if the mother senses that maybe this nurse won’t respond positively… she might feel intimidated and she wouldn’t ask” Counselling Psychologist  “A hostile attitude or dismissive attitude by the health care workers. If they are being ignored by the nurses… being yelled at… then she is not feeling comfortable to ask” – Counselling Psychologist  “Most government hospitals, if you say something, the nurses are quite dismissive, very dismissive… how are going to ask for something even if its pain medication” – Health Psychologist  “Most of the women especially in the rural settings would largely be illiterate or semi-illiterate and therefore when they are in the presence of who has been to school and has everything else and is in that uniform then they would obviously freeze and they would not ask and that’s quite a challenge” – Organisational Psychologist  “People already have that mentality that nurses in government hospitals are tough” – Health Psychologist  “The women don’t have the confidence that they were supposed to have because they feel like the person, I am talking to is educated, oh, the person I am talking to already knows what he or she is doing” – Developmental Psychologist  “It’s demand versus supply (in public hospitals) in the sense that you have very few health workers and a lot of mothers so you have fifty people behind you waiting for the same service provider… what about my friends who have been here since morning” – Health Psychologist  “There aren’t enough spaces where people can voice out their issues without being judged” – Developmental Psychologist  “If a mother is told that she cannot be helped because a service is not available, she will unlikely demand it the next time she comes.” Health Psychologist, KII. |

**Annex 3.6:** **Suggestions for the Demand Side**

| **Mothers** | **Providers** | **Psychologists** |
| --- | --- | --- |
| “Mothers should know all that is expected…and they need to be encouraged not to fear” (Mother, Kaluluma FGD  “You can give us leaflets of information about the things that we should be asking for” Mother, Kauluma FGD  “You have to come to the health center in time so that the doctor has also time to help you and listen to your requests.” Mother, Kauluma DGD | “During ANC health talks, women should be encouraged not to fear. They need to know they are not being rude when they demand. They are only claiming their rights. They need to be told what is demandable and what is not.” Health Worker, Kaluluma, Post Vignette Discussion on Observation of Care.  “They have to know the service charter... mothers should know if they come to the hospital what has to be demanded…the services that we can offer to them e.g., dishing leaflets… have posters to be pasted in the community and health centres so they know.” RDH Staff, KII. | “We should not underestimate the power of preconception counselling because it prepares psychologically the mothers. A lot of depression that comes in, it’s coming in because people were not ready for it. They were not well taught to say, okay, these are the changes that are going to be happening to your body.” Developmental Psychologist, KII.  “A multidisciplinary approach would help in these things…you can have the psychologist coming in talking to them about mental health. You can have someone who is very familiar with medical law coming in to talk to them about their rights as pregnant women.” Health Psychologist, KII.  “If there were those assertiveness trainings, the women will be well equipped, they will know for sure that what I am doing right now is right whether this person is more educated than me or not but what I am asking for is for my own good, is for my own health.” Developmental Psychologist, KII.  “Mental health is lacking… it’s a very important aspect, if it could be incorporated… mental health is not only about depression but even the issue of self-image and stuff like that.” Health Psychologist, KII.  “They (mothers) need psychotherapy… just to get the person to talk through the fear or the pain or whatever… just get the person to wrap their minds around about what they have gone through” – Counselling Psychologist  “Post-partum depression one is quite prevalent, and I feel that maybe if that could be explained during antenatal classes that sometimes you might go through ABC” – Health Psychologist  “Maybe using a guardian can as well help especially when maybe is just before delivery not during but maybe after” – Developmental Psychologist  “Encourage women to go to the same health facility, to develop familiarity and rapport, they are likely to feel comfortable enough to ask for certain services” – Counselling Psychologist |

**Annex 3.7: Suggestions for the Demand Side**

|  | | |
| --- | --- | --- |
| **Mothers** | **Providers** | **Psychologists** |
| “The way they conduct themselves…the way they walk…it should show that they have umunthu (‘humanity’). When someone is coming from that side, you should actually feel you can talk to them. That is nsangala zodutsa (‘friendliness by gesture/countenance’).” Mother, Santhe FGD.  “The doctor should not be far away…they should be present to check the woman regularly” (Mother, Kaluluma FGD)  “You can give us leaflets of information about the things that we should be asking for” Mother, Kauluma FGD  “We need the doctors to encourage us when we come to the clinic…that way we can be ready to ask for something.” Mother, Kauluma FGD  “We need the doctors to encourage us when we come to the clinic…that way we can be ready to ask for something.” Mother, Santhe FDG.  “The doctors also need to be punctual so that they have time for us.” Mother, Kaluluma FGD  “The doctor should not be short tempered.” Mother Santhe FDG | “During ANC health talks, health workers need to encourage women not to fear. Women need to know they are not being rude when they demand. They are only claiming their rights. They need to be told what is demandable and what is not.” Health Worker, Kaluluma, Post Vignette Discussion on Observation of Care.  “Health workers also have to be oriented…. They should be aware that if the mother comes with a demand, it’s not of her own making. They have been empowered to demand that…I think that will reduce misunderstandings.” Health Worker, Post Vignette Discussion on Exploring Demandable Services.  “Health care workers should be periodically oriented on rights of clients. They should also be oriented on respectful maternal care during preservice and in-service trainings.” RDH Staff, KII.  “Mmm…Frankly speaking…we are the one to change first so that the clients can ask questions. Other clients can’t even ask for any simple service that has omitted…We need to make them feel free.” Health Worker, Post Vignette Discussion on Observation of Care, Kaluluma.  "We need to praise the mothers when they demand and encourage them to ask questions…allow them to ask question and show interest when they have asked." Health Worker, Post Vignette Discussion on Observation of Care, Kaluluma. | “If they (health workers) communicate that…. they are warm, receptive and attentive to the patients that could also make the person comfortable… to ask for services” – Counselling Psychologist  “I will go to the health workers… maybe train them (on) the perception and the attitude… and communication” Health Psychologist  “Train health workers in/how to handle the women, what information to provide… what services are vital to the women” – Counselling Psychologist  “Training both the women and the health care providers on how to communicate” Counselling Psychologist  “Train both the mothers and health workers together; creating dialogue between mothers and health workers… having both groups there to discuss or air their grievance” – Counselling Psychologist  “Even adding on stress management skills because we know we understand that they are under stress…. So training them on how can they cope with that stress” – Developmental Psychologist  “Certain services are never explained... Explain why you are providing certain services.” – Organisational Psychologists  “If they (health workers) communicate that…. they are warm, receptive and attentive to the patients that could also make the person comfortable… to ask for services” – Counselling Psychologist |
